# CCHamide-2 Is an Orexigenic Brain-Gut Peptide in *Drosophila*

**DOI:** 10.1371/journal.pone.0133017

**Published:** 2015-07-13

**Authors:** Guilin R. Ren, Frank Hauser, Kim F. Rewitz, Shu Kondo, Alexander F. Engelbrecht, Anders K. Didriksen, Suzanne R. Schjøtt, Frederikke E. Sembach, Shizhong Li, Karen C. Søgaard, Leif Søndergaard, Cornelis J. P. Grimmelikhuijzen

**Affiliations:** 1 Center for Functional and Comparative Insect Genomics, Department of Biology, University of Copenhagen, DK-Copenhagen, Denmark; 2 Section of Cell and Neurobiology, Department of Biology, University of Copenhagen, DK-2100 Copenhagen, Denmark; 3 Genetic Strains Research Center, National Institute of Genetics, Mishima, Shizuoka, Japan; University of Cologne, GERMANY

## Abstract

The neuroendocrine peptides CCHamide-1 and -2, encoded by the genes *ccha1* and *-2*, are produced by endocrine cells in the midgut and by neurons in the brain of *Drosophila melanogaster*. Here, we used the CRISPR/Cas9 technique to disrupt the *ccha1* and *-2* genes and identify mutant phenotypes with a focus on *ccha-2* mutants. We found that both larval and adult *ccha2* mutants showed a significantly reduced food intake as measured in adult flies by the Capillary Feeding (CAFE) assay (up to 72% reduced food intake compared to wild-type). Locomotion tests in adult flies showed that *ccha2* mutants had a significantly reduced locomotor activity especially around 8 a.m. and 8 p.m., where adult *Drosophila* normally feeds (up to 70% reduced locomotor activity compared to wild-type). Reduced larval feeding is normally coupled to a delayed larval development, a process that is mediated by insulin. Accordingly, we found that the *ccha2* mutants had a remarkably delayed development, showing pupariation 70 hours after the pupariation time point of the wild-type. In contrast, the *ccha-1* mutants were not developmentally delayed. We also found that the *ccha2* mutants had up to 80% reduced mRNA concentrations coding for the *Drosophila* insulin-like-peptides-2 and -3, while these concentrations were unchanged for the *ccha1* mutants. From these experiments we conclude that CCHamide-2 is an orexigenic peptide and an important factor for controlling developmental timing in *Drosophila*.

## Introduction

The CCHamides are recently discovered arthropod neuropeptides, occurring in insects [1–8], crustaceans [9, 10], chelicerates [3, 11] and centipedes [12], suggesting that these peptides are generally occurring in arthropods. Insects normally have two CCHamide genes, one coding for CCHamide-1 and one coding for CCHamide-2, while the other arthropods only have one gene, suggesting that a gene duplication took place in the close ancestors of insects [3]. In *D*. *melanogaster*, CCHamide-1 has the structure SCLEYGHSCWGAHamide (where the two cysteine residues form a cystine bridge) and CCHamide-2 has the structure GCQAYGHVCYGGHamide (again with a cystine bridge) [3–5]. For both neuropeptides, their preprohormones have been identified and their receptors deorphanized [3, 5].

In this paper, we want to focus on *D*. *melanogaster* CCHamide-2. Using immunocytochemistry, we and others have previously localized CCHamide-2 in a specific population of endocrine cells in the gut [13, 14] and in a small group of neurons in the brain from *D*. *melanogaster* [13]. Using qPCR, we found that *D*. *melanogaster* CCHamide-2 mRNA was mainly produced in the gut (12 x higher mRNA concentrations in the gut compared to the brain), while CCHamide-2 receptor mRNA was mainly produced in the brain (45 x higher concentrations in the brain than in the gut) [13]. When normalized for the concentrations of CCHamide-2 mRNA, there is a 540 x higher CCHamide-2 receptor mRNA concentration in the brain than in the gut. These data led us to suggest that there exists a hormonal CCHamide-2 signaling pathway from the gut to the brain, probably related to feeding [13].

Little is known about the physiological actions of CCHamide-2. In the present paper, therefore, we investigate the role of CCHamide-2 in *D*. *melanogaster* by studying CCHamide-2 gene (*ccha2*) disruption mutants generated by the CRISPR/Cas9 technique [15, 16]. Our studies suggest that CCHamide-2 is an orexigenic (appetite-inducing) peptide, stimulating larval and pupal development probably through insulin signaling.

## Materials and Methods

### 2.1. Mutant and control fly strains and husbandry

**Fig 1:**
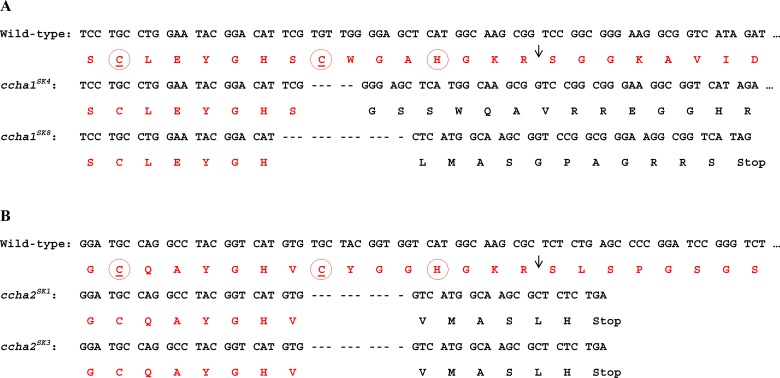
Nucleotide sequences and corresponding amino acid sequences around the deletions in two *ccha1* (A) and two *ccha2* (B) mutants. In the wild-type these nucleotide sequences code for the unprocessed CCHamide peptides, which are shown in red at the top of each panel. The black arrows in these red lines at the top indicate the initial cleavage steps in each prohormone, catalyzed by prohormone convertase [38]. A. Parts of the DNA sequences from the two *ccha1* mutants (*ccha1* ^*SK4*^ and *ccha1* ^*SK8*^) and the corresponding wild-type DNA sequence coding for CCHamide-1. Mutant *ccha1* ^*SK4*^ lacks 5 base pairs (bp), while mutant *ccha* ^*SK8*^ lacks 13 bp. Both deletions lead to a frameshift, so that no intact CCHamide-1 peptide can be produced. For example, while in the wild-type the two cysteine residues (underlined) form a cystine bridge, such ring structure can not be formed in the mutant peptides, because a second cysteine residue is lacking. Furthermore, while in the wild-type processing occurs between the KR and S amino acid sequence (arrow), followed by a conversion of the C-terminal G residue into a C-terminal amide [38], such posttranslational processings can not occur in the two mutants, due to the lack of the GKR amino acid sequence at these positions. The mutations, therefore, result in nonfunctional peptides that only have the N-terminal amino acid residues in common with wild-type CCHamide-1. B. Parts of the DNA sequences from *ccha2* mutants and their corresponding wild-type DNA sequences coding for CCHamide-2. The two mutants have identical 10 bp deletions that, again, cause a frameshift in the reading frame, resulting in the loss of the cystine bridge and the appropriate processing sites to yield functional peptides. Furthermore, the two mutants have a premature stop codon (TGA).

Flies were reared on corn meal (Nutri-Fly 116–12) at 25°C under a 12h light /12h dark photoperiod. *ccha1* and *ccha2* mutant flies were generated using the CRISPR/Cas9 technique [15, 16]. The following gRNA sequences were used: For *ccha1* GGAATACGGACATTCGTGTTGGG and *ccha2* GCCTACGGTCATGTGTGCTACGG, where the underlined 3-bp sequence is a Protospacer Adjacent Motif (PAM) sequence. For each gene, eight mutant candidates derived from male parents carrying *nos-Cas9* and *U6-gRNA* transgenes were screened by PCR and direct sequencing of the target region. In both cases, seven out of eight F1 offspring were found to carry an indel mutation. Two lines with a frameshift mutation were established for each gene (Fig 1). For many experiments described in this paper, we used heteroallelic combinations of the two *ccha1* mutants (*ccha1* ^*SK4*^ / *ccha1* ^*SK8*^) and of the two *ccha2* mutants (*ccha2* ^*SK1*^ / *ccha2* ^*SK3*^). Similarly, males from strain *y* ^*2*^ *cho* ^*2*^ *v* ^*1*^ crossed with females from strain *y* ^*1*^ *w* ^*1118*^ were used as a control, since the *ccha* mutants were generated using these genetic backgrounds.

### 2.2. The generation of rescued *ccha2* null mutants

To generate a *UAS-ccha2*, a PCR fragment containing the *ccha2* coding sequence was inserted into a pUAST vector: DNA fragments were PCR-amplified from cDNA using the primers shown in the Supplementary Information S1 Table. PCR was performed using the Hotstar-Taq Plus Master Mix Kit (Qiagen). Total RNA of wild-type flies was isolated using the RNeasy Kit (Qiagen). cDNA was synthesized and amplified from 200 ng total RNA using the SuperScriptIII First-Strand synthesis supermix (Invitrogen) and cloned into a pUAST vector. Transgenic flies were established by standard injection of the vector into *w* ^*1118*^ embryos by Bestgene Inc. (Chino Hills, CA-91709, USA).

To rescue the *ccha2* null mutant, we genetically combined a *UAS-ccha2* transgene with the *ccha2* ^*SK1*^ null allele (*UAS*-*ccha2; ccha2* ^*SK1*^ *)* and *arm-Gal4* with the *ccha2* ^*SK3*^ null allele (*arm-Gal4*;*ccha2* ^*SK3*^). The combination of *arm-Gal4*/*UAS-ccha2*; *ccha2* ^*SK1*^/*ccha2* ^*SK3*^ (*arm*>*ccha2*; *ccha2* ^*SK1*^/*ccha2* ^*SK3*^ *)* was obtained through standard genetic crosses. The *arm-Gal4* driver (CG11579) has a ubiquitously weak expression pattern.

### 2.3. Quantitative PCR

For *Drosophila* insulin-like peptide (DILP) mRNA measurements, early third instar larvae and stage P-5 pupae were used. Total RNA from these animals was isolated using the RNeasy Kit (Qiagen). This RNA was further purified using the DNA-free kit (Invitrogen) as described in the kit protocol. cDNA was synthesized and amplified from 500 ng purified total RNA, using the iScript advanced cDNA synthesis kit for RT-qPCR (BIO-RAD).

qPCR was performed in an Mx3005P instrument, using the Brilliant III Ultra-Fast SYBR qPCR master mix (Agilent). Primers were designed using CLC Main workbench 6.2 (see Supporting Information, S1 Table). Non-template (NT) and non-reverse transcriptase controls (NRT) were included to check for background and genomic DNA contaminations. *RpL11* (CG7726), *RpLP0* (CG7490), and *RpL32* (CG7939) were used as reference genes (see Supporting Information S1 Table for primers). Reference gene stability (requiring M≤1 and CV≤0.5; for a definition of M and CV see ref. [17]) were analysed by the qBASE-Plus program (Biogazelle NV, Zwijnaarde, Belgium).

For the qPCR measurements of *ccha2* mRNA we used our previously published primer set [13] and a new primer set published by a Japanese research group [18] (S1 Table). All other procedures were as for the DILP mRNA measurements described above.

### 2.4. Feeding assays in adult flies

We performed the Capillary Feeding (CAFE) assay to quantify food intake after starvation. The experimental set-up was described in [19]. Assays were carried at 16–18 o’clock using 4-d old adult flies pre-starved for 2 hours in tubes with 1% agar in a 25°C incubator. Male and female adult flies were measured separately. During the assay, the flies were fed on 5% sucrose containing 0.6 g/l Allura Red AC dye (Sigma-Aldrich), for 2 hours at 25°C without any disturbance. 10 tubes containing 4 flies each were measured in one day. These experiments were repeated on four subsequent days, each time using fresh 4-d old flies (n = 5). Tubes without flies were used as blank controls.

### 2.5. Larval feeding assays

The larval feeding assay based on the contraction rates of larval mouth hooks was described in [20]. Third instar larvae were transferred to the 1% agar plate covered with a 2% yeast solution. After an acclimatization of 30 sec, the number of mouth hook contractions were counted under the microscope for 30 sec. Each assay included 10 animals for each genotype, and the results were repeated in five independent experiments (n = 5). All experiments were conducted as blind tests.

The larval feeding assays based on yeast ingestion was described in [21, 22]. Third instar larvae were carefully transferred to an apple juice agar plate containing labeled yeast paste (labeled with AC Red) and incubated for 30 min. The larvae were subsequently washed and homogenized in 1 ml water, and the suspension passed through a 0.2 μm syringe filter. The absorbance of the eluate was measured at 509 nm. The amount of larval yeast intake was calculated from a standard curve made by serial dilutions of labeled yeast. 25 animals were grouped into five parallel samples of 5 animals (n = 5).

### 2.6. Developmental timing

Flies were allowed to lay eggs on apple juice agar plates for 4 hours. After 24-hour incubation, about 30 first instar larvae from each fly strain were transferred to tubes containing standard corn meal and incubated at 25°C. Paparium formation (stage P-2) was counted every 2–6 hours in three replicates each containing 20–30 animals. The results were plotted and analysed using the Graph Pad Prism6 program. The one-way ANOVA test was used for statistics.

### 2.7. Determination of adult body weight

Flies were raised on regular food at 25°C. 4-d old male and female files were anesthetized under CO_2_ exposure for 10min and immediately afterwards weighted as 10 independent groups of 10 flies (n = 10).

### 2.8. Wing size measurements

Wing sizes were determined as in [23]. Only the D-area was measured [23]. Right wings of 30 5-day old adult male and female flies were measured separately. Photographs of the wings were taken with a Zeiss Axio Zoom V16 stereomicroscope. Quantification of the area surface was performed with ZEN software (Zeiss).

### 2.9. Locomotion assay

Daily activity was assessed as described in [24]. Individual 4-d old male and female flies were collected under CO_2_ exposure and kept in a 12 hours light/dark cycle in a 25°C incubator, where the light turns on at 8 a.m. and off at 8 p.m. Furthermore they were supplied with food (5% sucrose in agar). Briefly, we monitored activities at 1 minute intervals using a *Drosophila* activity monitoring system (DAMS; TriKinetics, U.S.A.). Data from day-2 (6-d old flies) were chosen. In three independent experiments we measured each 32 flies for male and female mutants and male and female controls(n = 3). Control and experimental animals were processed strictly in parallel. We used the Graph Pad Prism6 program for statistical analyses.

### 2.10. Statistical analyses

Error bars indicate S.E.M. and the significance of the difference between data sets were calculated using the two-tailed Student’s t-test.

## Results

### 3.1. The *ccha1* and *-2* deletion mutant flies can not yield functional peptides

We sequenced the coding regions of the mutant *ccha1* and *-2* genes. For the *ccha1* disruption mutants, we found that the mutant named *ccha1* ^*SK8*^ had a 13 basepair (bp) deletion, lying within the coding region of the peptide, while mutant *ccha1* ^*SK4*^ had a 5 bp deletion (Fig 1A), also situated within the peptide coding region. Both deletions affect the DNA sequences coding for CCHamide-1 and cause a frameshift, leading to non-functional CCHamide-1 peptides, because the C-terminal halves of the peptides were exchanged by foreign sequences and a proper C-terminal processing site (GKR) was not available anymore (Fig 1A).

**Fig 2:**
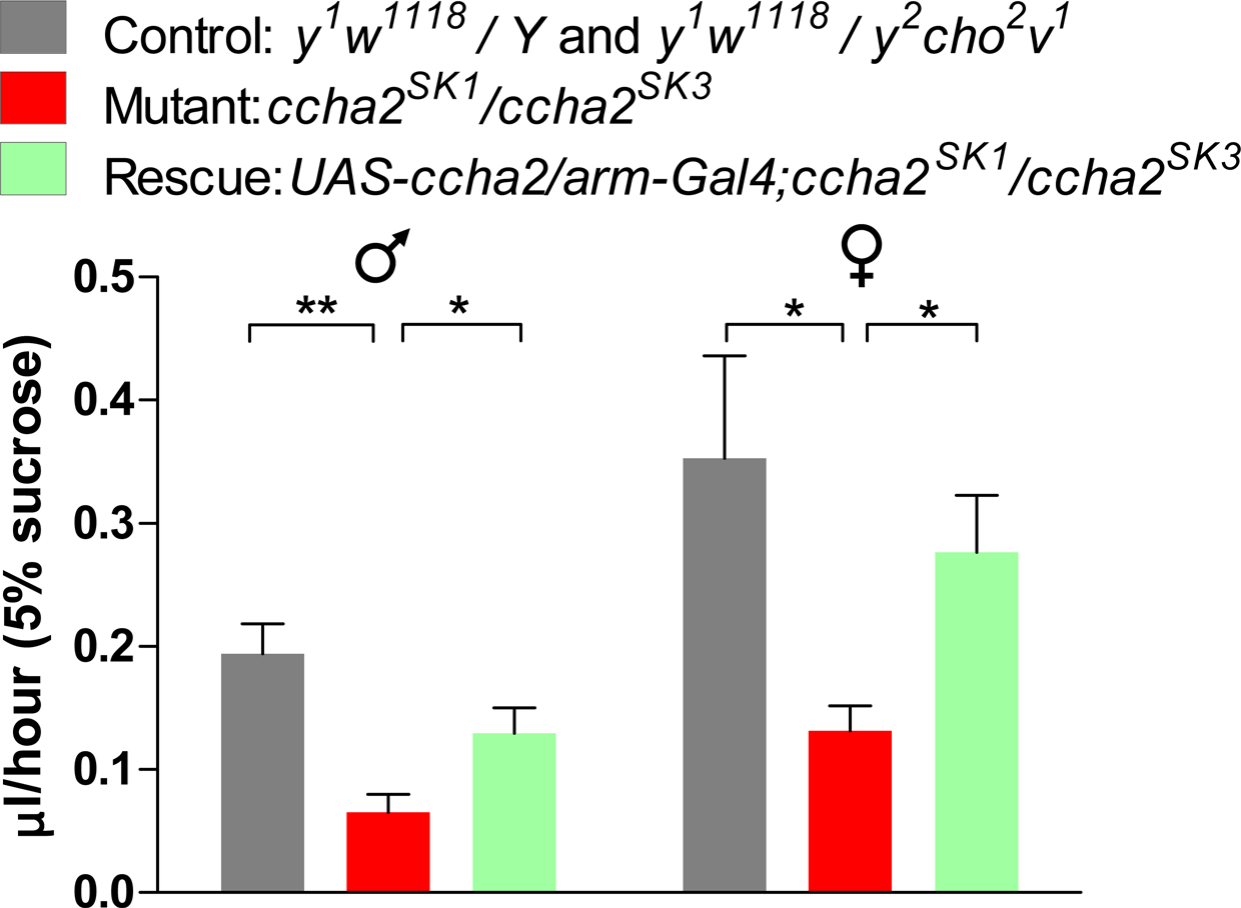
The capillary feeding (CAFE) assay for *ccha2* mutant male and female adult flies. Each data point was an average from the results obtained from 10 tubes containing 4 flies each. The experiments were repeated five times, each time with fresh animals. The controls are indicated by black bars, the mutants (*ccha2* ^*SK1*^/*ccha2* ^*SK3*^) by red bars, while the rescued mutants are indicated by green bars. The mutant male flies have 30% feeding activity left compared to the controls (n = 5; t-test, ** p≤0.01), while the mutant female flies have 37% feeding activity left compared to the controls (n = 5; t-test, * p≤0.5). The rescued male and female *ccha2* mutants more than doubled their feeding activities compared to the *ccha2* null mutants (*p≤0.5). The vertical bars represent S.E.M.

The *ccha2* mutants named *ccha2* ^*SK3*^ and *ccha2* ^*SK1*^ have identical deletions of 10 bp that, again, affect the C-terminal half of the CCHamide-2 peptide (Fig 2B). Also here, the deletions cause frameshifts leading to non-functional peptides (Fig 1B).

Because the *ccha1* mutants did not show phenotypes different from the wild-type in our behavioral assays, we will focus on the properties of the *ccha2* mutants in the Results section.

### 3.2. CCHamide-2 is an orexigenic peptide in adult flies

In a Capillary Feeding (CAFE) assay, adult flies containing the disrupted *ccha2* gene have a significantly reduced food intake, which is 70% reduced in male and 63% reduced in female flies compared to the controls (red bars in Fig 2). These results suggest that in wild-type animals, CCHamide-2 stimulates food intake, or in other words that CCHamide-2 is an orexigenic peptide.

The feeding phenotype of adult *ccha2* mutants can be rescued (to about 80% of the feeding activities of the controls; see green bars in Fig 2) by reintroducing the intact *ccha2* gene, showing that the feeding deficiency of the *ccha2* mutants was solely due to the deletion of the *ccha2* gene.

### 3.3. CCHamide-2 stimulates locomotion related to feeding in adult flies

**Fig 3:**
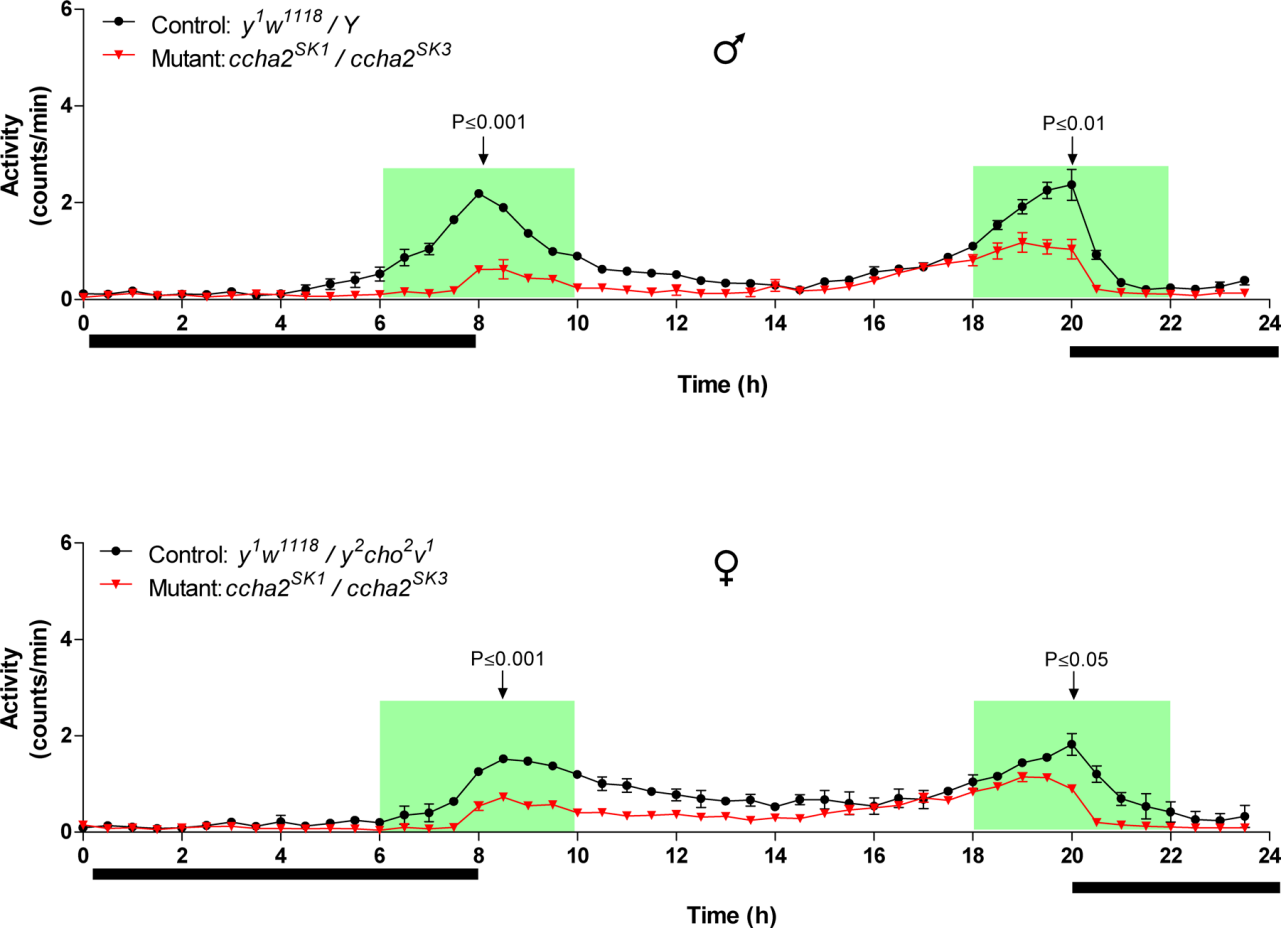
Circadian activities of the control (black lines) and CCHamide-2 mutants (red lines). The activities were measured using a *Drosophila* activity monitor that monitors one-dimensional locomotion of single flies. Light is switched on at 8 a.m. and switched off at 8 p.m. The upper panel gives the activities of 6-d old male, and the lower panel of 6-d old female flies. The data points represent the average of three independent experiments containing 32 flies each (n = 3). The vertical bars represent S.E.M. When no vertical bars are visible, they are smaller than the symbols used. The green areas highlight time periods around 8 a.m. and 8 p.m., where the activity differences between mutants and wild-type were especially significant. These periods coincide with the normal feeding periods of wild-type *Drosophila* [25]. The arrows indicate significant activity differences between mutants and controls at 8 a.m. and 8 p.m. P-values are between p≤0.001 and p≤0.05.

We next asked whether reduced feeding would affect locomotion in *ccha2* mutants. Using an automated one-dimensional *Drosophila* activity monitor (DAM; TriKinetics), where the circadian locomotor activities of single adult flies can be measured, we found that both adult male and female mutant flies had significantly reduced locomotion (Fig 3). Compared to the control (wild-type), these reduced locomotor activities were especially prominent around the time periods, where the light is switched on (8 a.m.) and off (8 p.m.) (highlighted by the green area in Fig 3). At 8 a.m. the mutants showed locomotor activities that were 72% reduced in males and 60% reduced in female flies compared to wild-type (Fig 3). At 8 p.m., these numbers were 56% and 48%. Both controls and mutants have increased locomotor activities around 8 a.m. and 8 p.m. It is known that these periods are used by fruitflies for foraging and food intake [25]. We conclude, therefore, that CCHamide-2 specifically stimulates those locomotor brain circuits in wild-type flies that are part of their foraging and feeding behavior.

### 3.4. CCHamide-2 is also an orexigenic peptide in larvae

**Fig 4:**
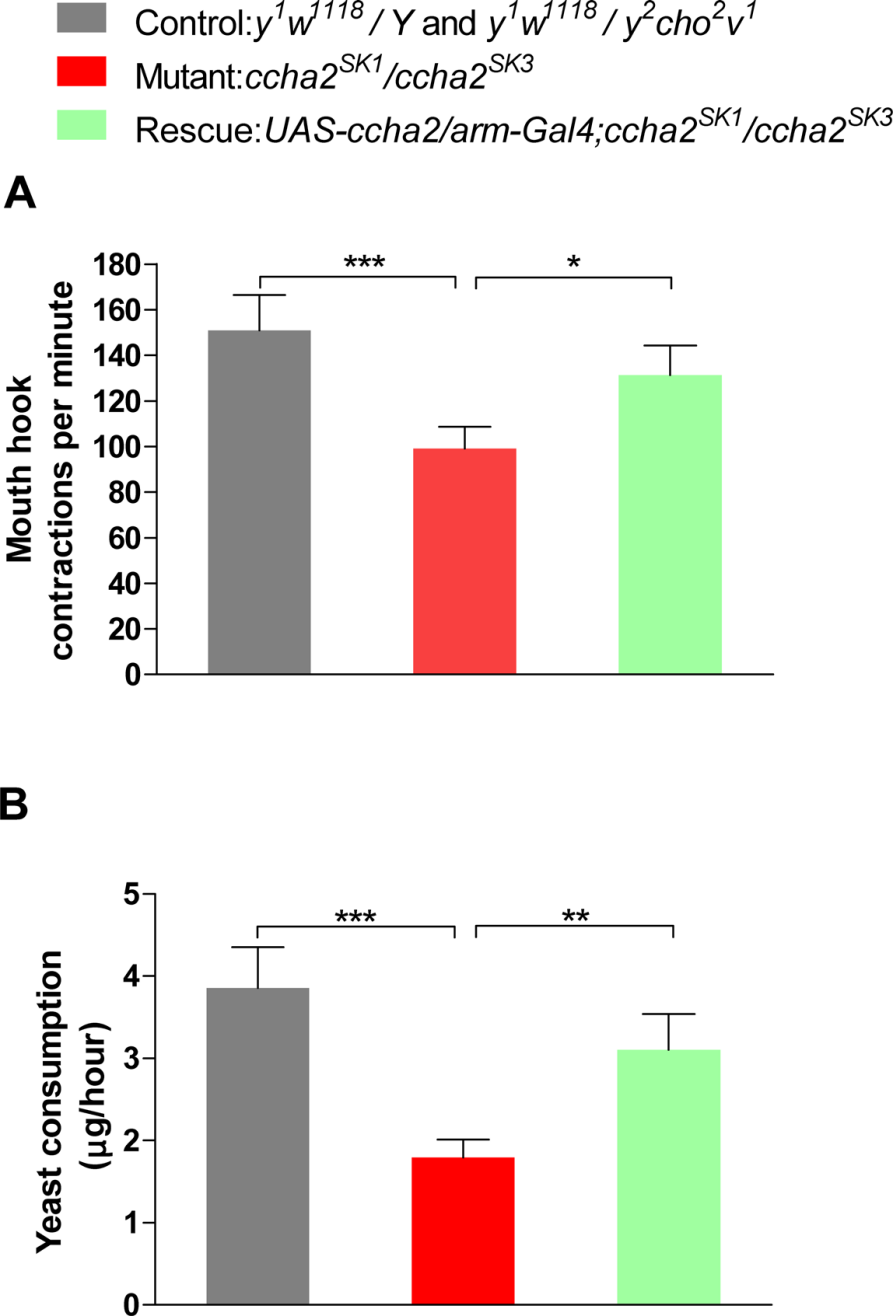
Larval feeding assays for control flies (black bars), *ccha2* null mutants (red bars) are rescued *ccha2* mutants (green bars). A. Larval feeding assay measuring the frequency of mouth hook contractions of third instar larvae feeding on agar covered with a 2% yeast solution. The *ccha2* null mutants have 63% of their feeding activity left compared to the controls (n = 5; t-test. *** p≤0.001). The rescued mutants restored their feeding activity to a level which is 86% of the control activity. The difference between *ccha2* null mutants and rescued mutants is significant (t-test, * p≤0.5). B. A different larval feeding assay, measuring the amount of ingested color-labelled yeast per hour. The *ccha2* null mutants have 43% of their feeding activities left compared to controls (n = 5; t-test, *** p≤0.001). The rescued mutants restored their feeding activity to 78% of the controls. The difference between *ccha2* null and rescued mutants is significant (t-test, ** p≤0.01).

We also investigated the feeding behavior in larvae, using two different larval feeding assays (Fig 4). In one assay, we measured the frequencies of the mouth hook contractions of early third instar larvae feeding on a 2% yeast solution (Fig 4A) and found that the *ccha2* mutants (red bar in Fig 4A) had a 40% reduced feeding activity compared to the controls (black bar in Fig 4A). This impaired feeding activity could be rescued to 86% of the control feeding values (green bar in Fig 4A) by reintroducing the *ccha2* gene into the *ccha2* null mutant larvae.

In another larval feeding assay we measured the speed of yeast consumption (Fig 4B) and found that the *ccha2* mutants (red bar in Fig 4B) had a 57% reduced feeding rate compared to controls (black bar in Fig 4B). Also here, the mutant phenotype could be rescued by introducing the *ccha2* gene into the *ccha2* null mutant larvae (green bar in Fig 4B). Again, these results suggest that the feeding deficiencies of the larvae was solely due to the deletion of the *ccha2* gene.

### 3.5. CCHamide-2 stimulates larval development

**Fig 5:**
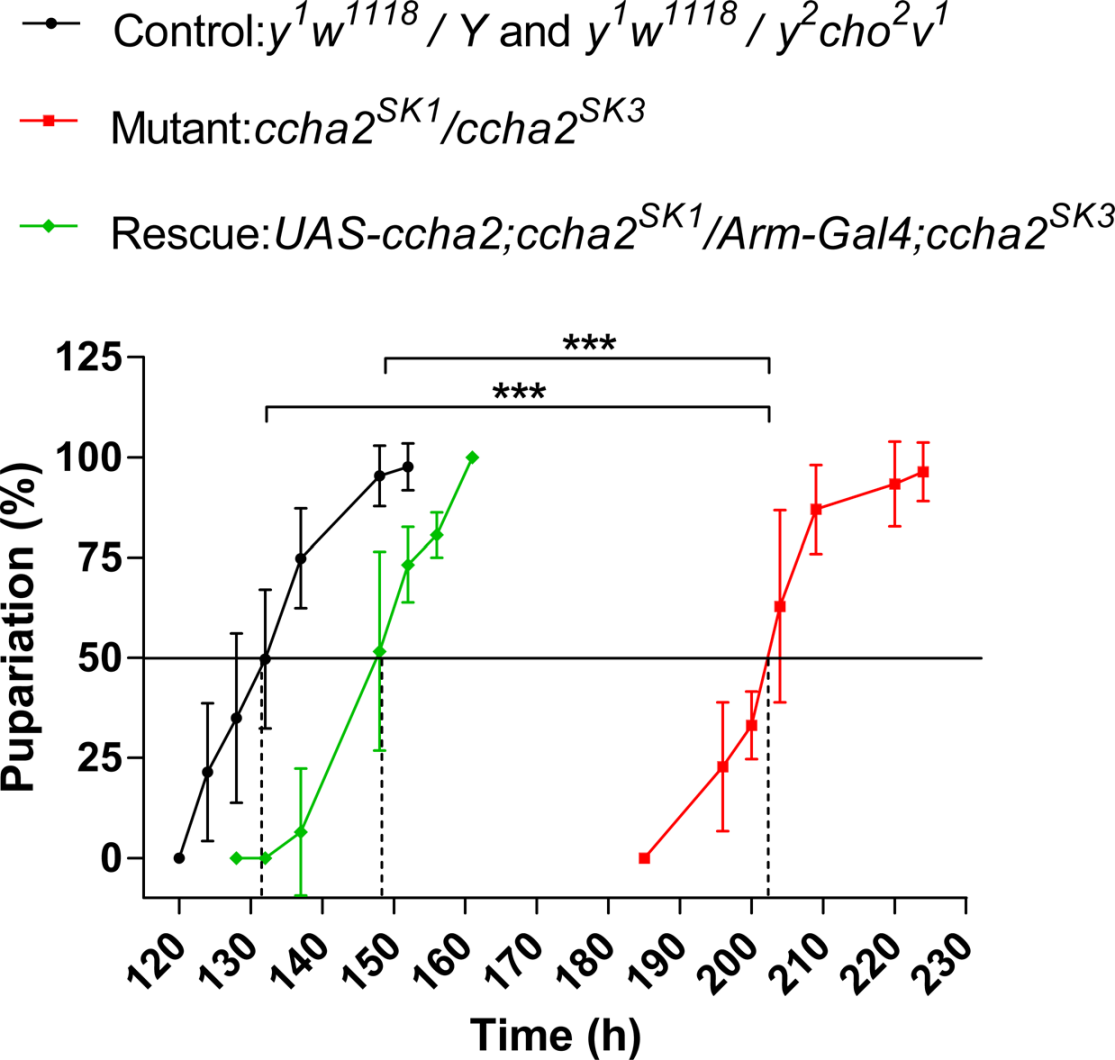
Pupariation time points of *ccha2* mutants compared to control. The horizontal line parallel to the abscissa indicates 50% of the animals having undergone pupariation. The vertical stippled lines indicate the time points, where 50% of the experimental animals have pupariated. Control animals (indicated by a black line) pupariated (pupal stage P-2) at 132 hrs after egg laying Homozygous mutants (indicated by a red line) pupariated at 202 hrs after egg laying and were, therefore, 70 hrs delayed compared to controls. Furthermore, *ccha2* mutants rescued by re-introducing the *ccha2* gene (indicated by a green line) pupariated at 148 hrs and were, thus rescued by 80%. The data points represent the average of five independent experiments, containing 15–25 animals each. The vertical bars represent S.E.M. The differences between control and *ccha2* mutants, and between *ccha2* mutants and rescued mutants are stastically significant (one-way ANOVA test, p≤0.001).

Adequate food intake is important for proper larval development. We, therefore, investigated the *ccha2* mutants for their developmental timing, i.e. the time needed to develop from a freshly deposited egg to a pupa. Fig 5 shows that control *D*. *melanogaster* (black line in Fig 5) needed about 130 hrs (at 25°C) to pupariate. In contrast, the mutant flies that lacked the intact CCHamide-2 gene (red line in Fig 5) were severely delayed and needed about 200 hrs for this process, which was a delay of about 70 hrs (Fig 5). Mutant *ccha* flies, where the *ccha2* gene was reintroduced (green line in Fig 5), pupariated at about 150 hrs, meaning that they had been rescued by about 75%, which is the same number seen in the feeding assays (green bars in Fig 2 and Fig 4).

We also measured heterozygous mutant flies that had one intact *ccha2* allele, while the other allele was disrupted. These flies pupariated at about 160 hrs, i.e. at a time point intermediate between the pupariation times of the control and the homozygous mutants (S1 Fig, Supplementary Information).

Interestingly, the developmental timing of fly mutants lacking the intact *ccha1* gene (Fig 1B) is not different from that of the control flies (S2 Fig), showing that the CCHamide-1 peptide must have a physiological function in the flies that is quite different from that of CCHamide-2.

### 3.6. The involvement of insulin-like peptides in the phenotype of the CCHamide-2 deletion mutants

**Fig 6:**
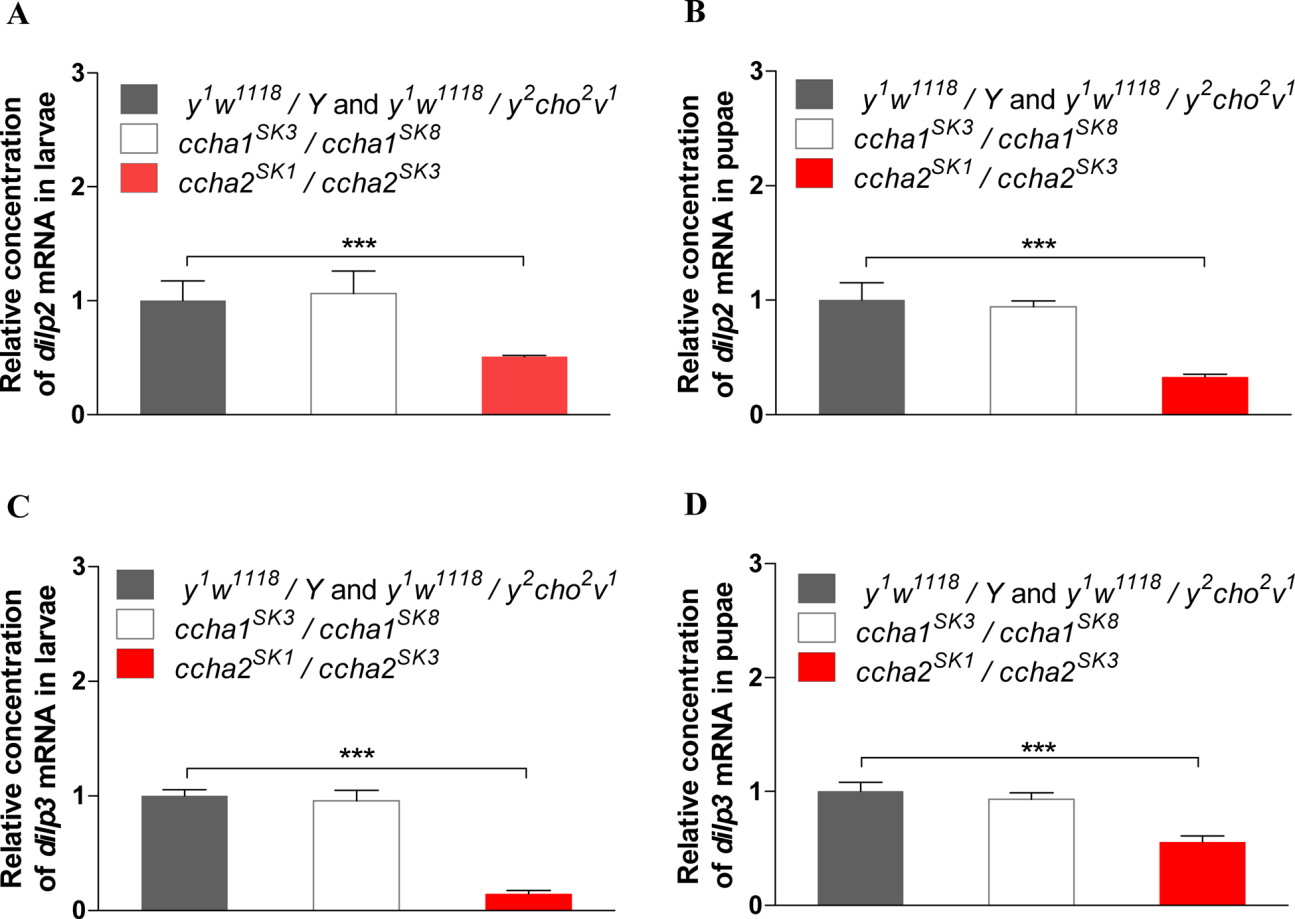
qPCR of *Drosophila* insulin-like peptide (DILP) gene expressions in third instar larvae and pupae of ccha1 and -2 null mutants and wild-type animals. Control animals are indicated by black bars, *ccha1* mutants are indicated by white bars, *ccha2* mutants are indicated by red bars. The vertical bars represent S.E.M. (n = 3). Thirty animals were used in each measurement (2 technical replicates; 3 biological replicates). A. In larval *ccha2* mutants, *dilp2* gene expression is reduced by about 50% (t-test, *** p≤0.001), while in larval *ccha1* mutants there is no reduction compared to wild-type. B. In pupal *ccha2* mutants (pupal stage P-5), *dilp2* gene expression is reduced to 35% of the wild-type values (t-test, *** p≤0.001), while there is no reduction in *ccha1* mutants. C. In larval *ccha2* mutants, *dilp3* gene expression is reduced to 20% of the wild-type values (t-test, *** p≤0.001), while there is no such downregulation in *ccha1* mutants. D. In pupal *ccha2* mutants (stage P-5), the *dilp3* gene expression is downregulated to about 50% of the wildtype values (t-test, *** p≤0.001), while there is no significant downregulation in the pupal *ccha1* mutants.

A strongly delayed developmental timing in larvae of *D*. *melanogaster* is often associated with reduced insulin signaling [26]. Therefore, we carried out qPCR of the *D*. *melanogaster* insulin like peptide (DILP) mRNA’s: DILP-2 and DILP-3. Fig 6 shows that both third-instar larvae (Fig 6A) and pupae (Fig 6B) of the *ccha2* mutants have strongly reduced expressions of the *dilp2* gene. The expression of the *dilp3* gene is likewise strongly reduced, both in third-instar larvae (Fig 6C) and pupae (Fig 6D).

Interestingly, *dilp2* and *dilp3* gene expressions were normal in the mutants that lacked the intact *ccha1* genes (Fig 6), which is in agreement with our findings that there were no developmental delays in these mutants (S2 Fig).

**Fig 7:**
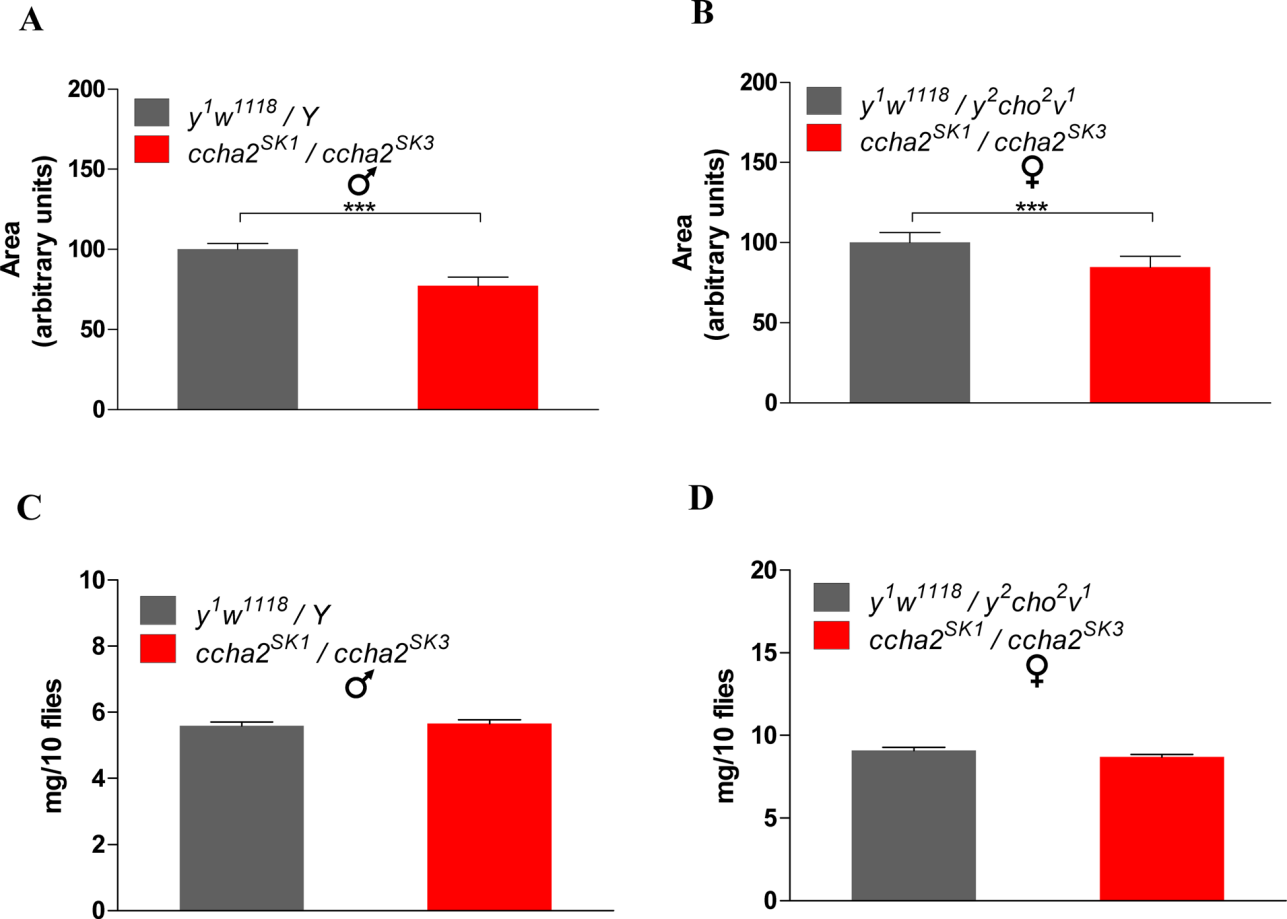
Wing size and adult fly weight in *ccha2* disruption mutants compared to wild-type. Wild-type animals are indicated by black bars, *ccha2* mutants are indicated by red bars. Vertical bars represent S.E.M. (n = 30). A. The wing surface of male *ccha2* mutants is 22.7% reduced compared to wild-types (n = 30; student t-test, *** p≤0.001). B. The wing surface of female mutants is 15.2% reduced compared to wild-types (n = 30; student t-test *** p≤0.0001). C. There is no significant weight difference between male *ccha2* mutants and wild-types (n = 100). D. There is no significant weight difference between female *ccha2* mutants and wild-types (n = 100).

Normal wing size is dependent on sufficient nutrition, a process that, again, is mediated by insulin signaling [27]. We found that both the male and female mutants lacking the intact *ccha2* gene had significantly reduced wing surfaces (23% reduced in males, 15% reduced in females compared to wild-type; Fig 7A and 7B).

Also, the *D*. *melanogaster* body weight is dependent on sufficient nutrient intake and under the control of insulin signaling [28, 29]. We expected, therefore, that the body weights of the male and female fly mutants lacking the intact ccha2 gene also would be reduced. This, however, was not the case (Fig 7C and 7D).

### 3.7. The role of the fat body as a source of CCHamide-2

**Fig 8:**
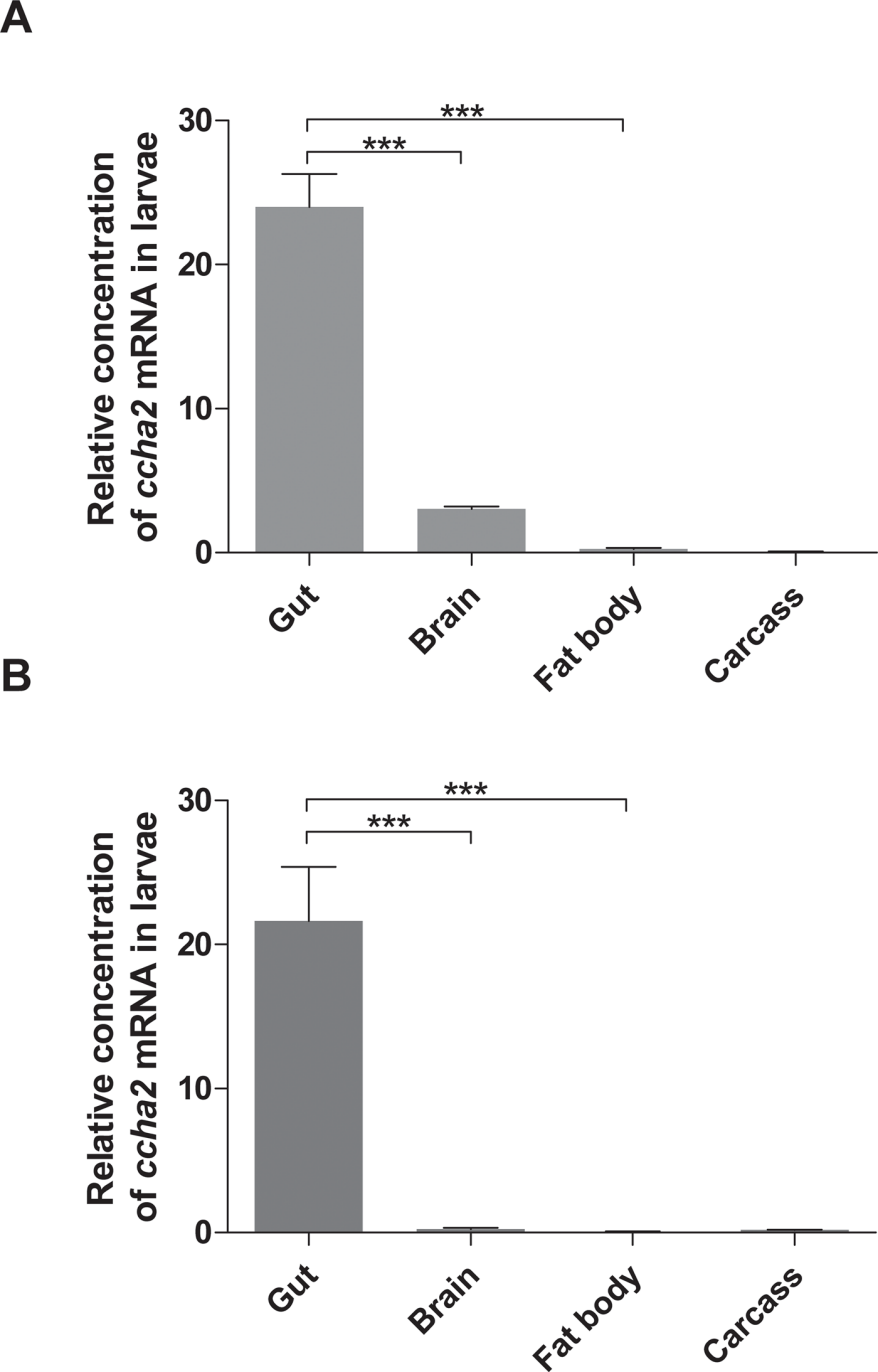
Expresion of the *ccha2* gene in different organs of mid third instar *D*. *melanogaster* larvae (92 hrs after egg laying). Two primer sets were used for qPCR: One primer set previously applied by us [13] and one primer set used by Sano et al. [18] (S1 Table). (A) qPCR results using the primer set described by us [13]. (B) qPCR results using the primer set described by Sano et al. [18]. It is clear from both experiments that the gut is the major source of *ccha2* mRNA, while the fat body is virtually devoid of *ccha2* mRNA (n = 3; student t-test *** p≤0.001).

After our paper had been submitted (21st February 2015) another paper by Sano et al. [18] was published (28th May 2015) also describing *ccha2* null mutants from *D*. *melanogaster*. In their paper Sano et al. found that the larval fat body was the major source of CCHamide-2 [18], which was in strong contrast to our earlier findings [13] that CCHamide-2 was mainly produced by the gut and, to a lesser extent, by the brain in third instar larvae. To settle this question, we prepared gut, brain, fat body, and carcass (rest of the body) in three independent experiments from third instar larvae and synthesized cDNA from these tissues. The qPCR results using our own set of primers [13] (Fig 8A) and the primers described by Sano et al. [18] (Fig 8B) clearly show that the *ccha2* gene is mainly expressed in the gut and to a lesser extent in the brain, while the fat body and carcass only have very low or no expression of *ccha2*, thus confirming our previous measurements [13].

## Discussion

The results of our CAFE assays on adult flies (Fig 2) and the larval feeding assays (Fig 4) suggest that CCHamide-2 must have a stimulatory effect on food intake in wild-type flies, because the feeding activities of mutants that lack the intact *ccha2* gene are remarkably reduced. The capillaries that contain the liquid food in the CAFE assay set-up, however, can only be reached by flying, making the outcome of the CAFE assay also dependent on the mobility of the animals. Also in larvae feeding is not independent from moving.

We, therefore, measured the adult *ccha2* mutants in a *Drosophila* activity monitor (Fig 3), which indeed showed that the mutants had a strongly reduced mobility compared to wild-type, which in this case was not measured as flying, but as walking in a one-dimensional direction. Remarkably, these mobility differences between mutants and wild-type were especially evident around 8 a.m. and 8 p.m., which are time periods, where adult wild-type animals are more active due to their innate feeding behavior [25]. At other time intervals, however, for example between 2 p.m. and 6 p.m., there were hardly any activity differences between mutants and wild-type (Fig 3). Mutant activities at 6 p.m. were even higher than at 8 a.m., which clearly shows that mutants are not generally less active that wild-type, but only less so in relation to feeding and foraging. These data combined with the results from Figs 2 and 4 suggest to us that CCHamide-2 stimulates feeding motivation and food seeking behavior, i.e. that it is an orexigenic neuropeptide or peptide hormone.

It is unlikely that the primary defect in the *ccha2* mutants is a dysfunction of the muscular system, because the CCHamide-2 containing endocrine cells and neurons in wildtype larvae do not directly innervate the skeletal muscles [13, 14]. Also the mouth hooks, which are used by larvae for food ingestion (Fig 4A) are not directly innervated by CCHamide-2 neurons [13, 14]. We conclude, therefore, that the primary defect in the *ccha2* mutants is a decreased feeding motivation.

The other experiments described in Figs 5 and 6 are in our eyes just a consequence of reduced feeding motivation of the mutants. Reduced food-intake leads to reduced insulin (DILP-2 and -3) signaling in larvae (Fig 6), leading to a 70-hr delay in reaching the developmental check point for pupariation (Fig 5).

Insulin stimulates growth and orchestrates the growth rates of the various organs in developing *D*. *melanogaster* in such a way that these organs reach their appropriate sizes (i.e. proportional to the body size) in the newly hatched adult flies [26–29]. The involvement of lowered insulin concentrations in the above-mentioned developmental delay (Fig 5), therefore, is further supported by our findings that the *ccha2* mutants have significantly smaller wings than the wild-type (Fig 7A and 7B). We would also expect that the mutants had lower body weights than the wild-type. This, however, turned out not to be the case (Fig 7C and 7D), a result that we do not understand, so far.

**Fig 9:**
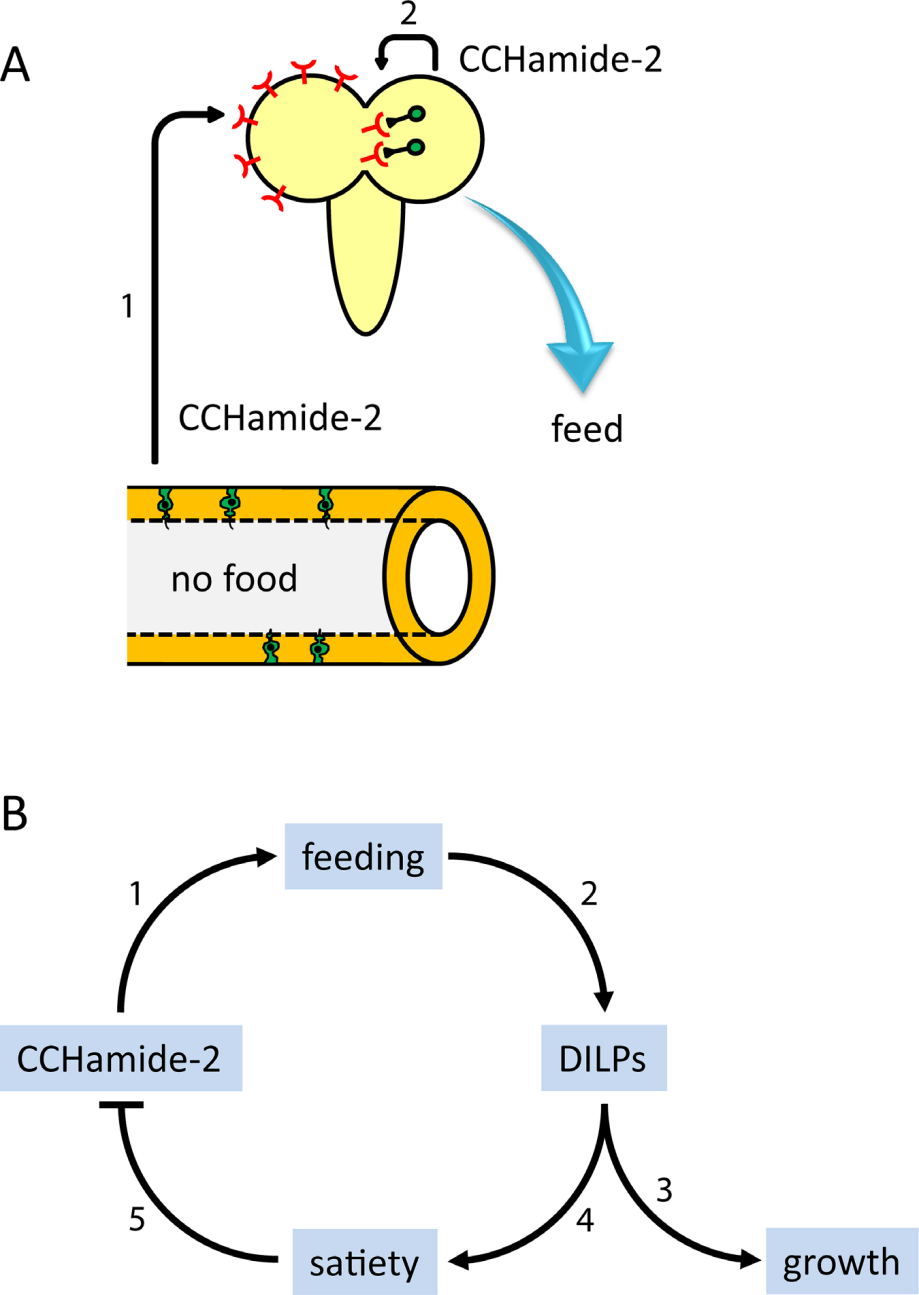
Hypothetical model for the actions of CCHamide-2 in *D*. *melanogaster*. A. When the lumen of the midgut (lower part of Fig 8A) is devoid of nutrients, the CCHamide-2 containing endocrine cells of the gut wall (highlighted in green) signal this information to the brain by releasing CCHamide-2 into the circulation (arrow 1). After binding to its brain receptors, CCHamide-2 induces foraging and feeding behavior. In addition to this long-distance CCHamide-2 signaling pathway, there is a short distance CCHamide-2 signaling pathway (arrow 2), where a small group of CCHamide-2 neurons in the brain (highlighted in green) also innervate the motor circuits underlying foraging and feeding. We hypothesize that these neurons might perhaps directly monitor the nutrients in the circulation. B. A flow diagram of the proposed sequence of events after CCHamide-2 has induced feeding (step 1; see also Fig 8A). Feeding induces the release of DILPs (Step 2; see refs 26, 31, 32). DILPs stimulate growth (step 3; see refs 26, 28), but also induce satiety (step 4; see ref 30, 31, 33). It is assumed that satiety blocks the release of CCHamide-2 and other orexigenic neuropeptides.

Figs 2 and 4 show that a loss of CCHamide-2 results in a strong reduction of feeding activity in both larvae and adults. Furthermore, Fig 6 shows that the loss of CCHamide-2 also leads to a strong reduction of *dilp2* and *dilp3* expression. There are two possibilities to explain these effects. First (possibility-1), the loss of CCHamide-2 → reduced feeding → reduced DILP expression. Or (possibility-2), the loss of CCHamide-2 → reduced DILP expression → reduced feeding. We strongly favour the first sequence of events, because it is known from the literature that DILPs induce satiety and inhibit feeding [30, 31], which is in conflict with the second possibility. Fig 9B, therefore, illustrates possibility-1.

Ida and co-workers [5] injected CCHamide-2 into living blowflies and observed an increased number of the proboscis extension reflexes when sucrose solutions were offered to these treated animals compared to ringer-injected flies. Their conclusion was that CCHamide-2 stimulates the feeding motivation in flies. This conclusion fits very well with our own conclusion that CCHamide-2 is an orexigenic peptide in *D*. *melanogaster*.

Using qPCR we previously found that most CCHamide-2 peptide mRNA was expressed in the gut, while most receptor mRNA was expressed in the brain [13]. Moreover, using immunocytochemistry, we found numerous CCHamide-2 containing endocrine cells in the midgut and a small group of CCHamide-2 containing neurons in the brain of *Drosophila* [13]. From these findings we postulated a model, where there are two CCHamide-2 signaling pathways in *D*. *melanogaster* (i) a long distance (hormonal) pathway from the gut to the brain, and (ii) a short-distance (paracrine or synaptic) pathway within the brain (Fig 9A). In how far can this previous model be reconciled with the new findings from our present study? We feel that these new findings can easily be accommodated in a somewhat extended model (Fig 9A), where the CCHamide-2 producing endocrine cells in the gut wall sense the absence of food in the gut lumen and transmit this information to the brain by releasing CCHamide-2 into the circulation (arrow #1 in Fig 9A). After binding to its specific receptors in the brain, CCHamide-2 starts a foraging and feeding behavior in the animal. In addition to this long-distance signaling from the gut to the brain, there must be a short-distance signaling within the brain (arrow #2 in Fig 9A), because the brain also contains about 40 CCHamide-2 immunoreactive neurons [13]. We speculate that these neurons might also be involved in the foraging and feeding response, perhaps directly measuring the carbohydrate concentration in the hemolymph and, when low, stimulating the same neuronal circuits that control the foraging and feeding behavior as the ones that are targeted by arrow #1 in Fig 9A.

Fig 9B gives the consequences of CCHamide-2 induced feeding, where step-1 is experimentally supported by the experiments given in Figs 2 and 4; step-2 by the experiments given in Fig 6 and data from the literature [26, 32, 33]; step-3 by the experiments from Fig 5 and data from the literature [26–29]; and step-4 by data from the literature [30, 31]. We assume that the induction of satiety coincides with a blockage of the release of CCHamide-2 (step-5).

In addition to CCHamide-2, other neuropeptides in *Drosophila* are known to be orexigenic, such as short neuropeptide F (sNPF) and, perhaps, neuropeptide F (NPF) [34, 35]. Also these peptides are produced by both endocrine cells in the gut and by neurons in the brain [35, 36]. The residual feeding activities in the CCHamide-2 deficient mutants (Figs 2 and 4) and, in general, their survival (Fig 5), is probably due to these additional orexigenic peptides, acting in parallel to the CCHamide-2 system. In addition to orexigenic peptides, there are also anorexigenic peptides in *Drosophila*, but only one such peptide has been functionally identified, namely allastostatin-A [37]. Like the CCHamide-2, sNPF, and NPF peptides, also the allastostatin-A peptides are brain-gut peptides [36].

*Drosophila* has turned out to be a versatile model for studying various human diseases and, in principal, could also be a valuable model for understanding obesity and other diseases related to the dysregulation of feeding and satiety in humans. As explained above, only few neuropeptides in *Drosophila* have been shown to be involved in feeding. The present addition of a novel orexigenic peptide, CCHamide-2, to the *Drosophila* “feeding toolkit”, therefore, is an important step forward in our understanding of feeding in *Drosophila* and other insects.

After our paper had been submitted to this journal (21st February 2015), another paper was published (28 May 2015) by Sano et al. [18], where it was proposed that the larval fat body from *D*. *melanogaster* was the major source of CCHamide-2 and that CCHamide-2 released from the fat body would control DILP release from the larval brain. We were, however, unable to reproduce their experiments (Fig 1A from ref [18]) showing that 97% of the larval *ccha2* mRNA occurs in the fat body. Instead, we found that most *ccha2* mRNA occurs in the gut and a minor portion in the brain, while the fat body contains little or no mRNA (Fig 8A). Similar results were obtained when we used the primer set that was described by Sano et al. [18] (Fig 8B). Therefore, it is unlikely that the larval fat body uses CCHamide-2 as a nutrient-responsive hormone for regulating DILP secretion in the brain [21]. In addition, we noticed that the primer set used by Sano et al. [18] for qPCR measurements of *ccha2* mRNA did not correspond to *ccha2* but to *ccha1* cDNA. This finding questions the results from all *ccha2* mRNA measurements described by Sano and co-workers [18].

## Supporting Information

S1 Fig Pupariation time points of *ccha2* mutants compared to control. The long horizontal line parallel to the abscissa indicates 50% of the animals having undergone pupariation. The vertical stippled lines indicate the time points, where 50% of the experimental animals have pupariated. Control animals (indicated by black lines) pupariated at about 130 hrs after egg laying. Homozygous mutants (indicated by red lines) pupariated at about 200 hrs after egg laying and were, thus, 70 hrs delayed compared to controls. Heterozygous mutants (indicated by blue lines) that contained one intact and one deleted *ccha2* allele had an intermediate pupariation time point (160 hrs). The data points represent the average of three independent experiments, containing 20–30 animals each. The vertical bars represent S.E.M. The differences between control, homo- and heterozygous mutants are statistically significant (ANOVA test, p ≤ 0.0001).
(PDF)

S2 Fig Pupariation time points of the *ccha1* disruption mutants and controls. There is no difference between the time points, where the *ccha1* mutants pupariate (127 hrs) and that of the control animals (ANOVA test, no significant difference).
(PDF)

S1 Table Primer sequences used in qPCR. (PDF)
