# Supplementary figures and images for: CCHamide-2 Is an Orexigenic Brain-Gut Peptide in Drosophila
Source: PLoS One. 2015 Jul 13;10(7):e0133017. doi: 10.1371/journal.pone.0133017 (PMC4500396; doi:10.1371/journal.pone.0133017)

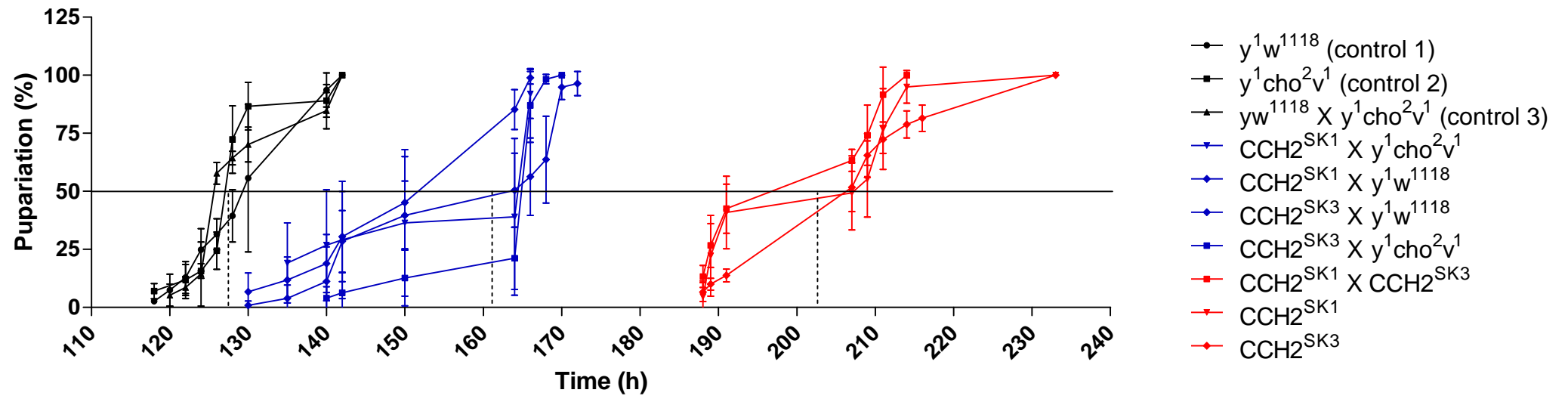

Fig. S1.

Supplement: S1 Fig — The long horizontal line parallel to the abscissa indicates 50% of the animals having undergone pupariation. The vertical stippled lines indicate the time points, where 50% of the experimental animals have pupariated. Control animals (indicated by black lines) pupariated at about 130 hrs after egg laying. Homozygous mutants (indicated by red lines) pupariated at about 200 hrs after egg laying and were, thus, 70 hrs delayed compared to controls. Heterozygous mutants (indicated by blue lines) that contained one intact and one deleted ccha2 allele had an intermediate pupariation time point (160 hrs). The data points represent the average of three independent experiments, containing 20–30 animals each. The vertical bars represent S.E.M. The differences between control, homo- and heterozygous mutants are statistically significant (ANOVA test, p ≤ 0.0001). (PDF) [file pone.0133017.s001.pdf]

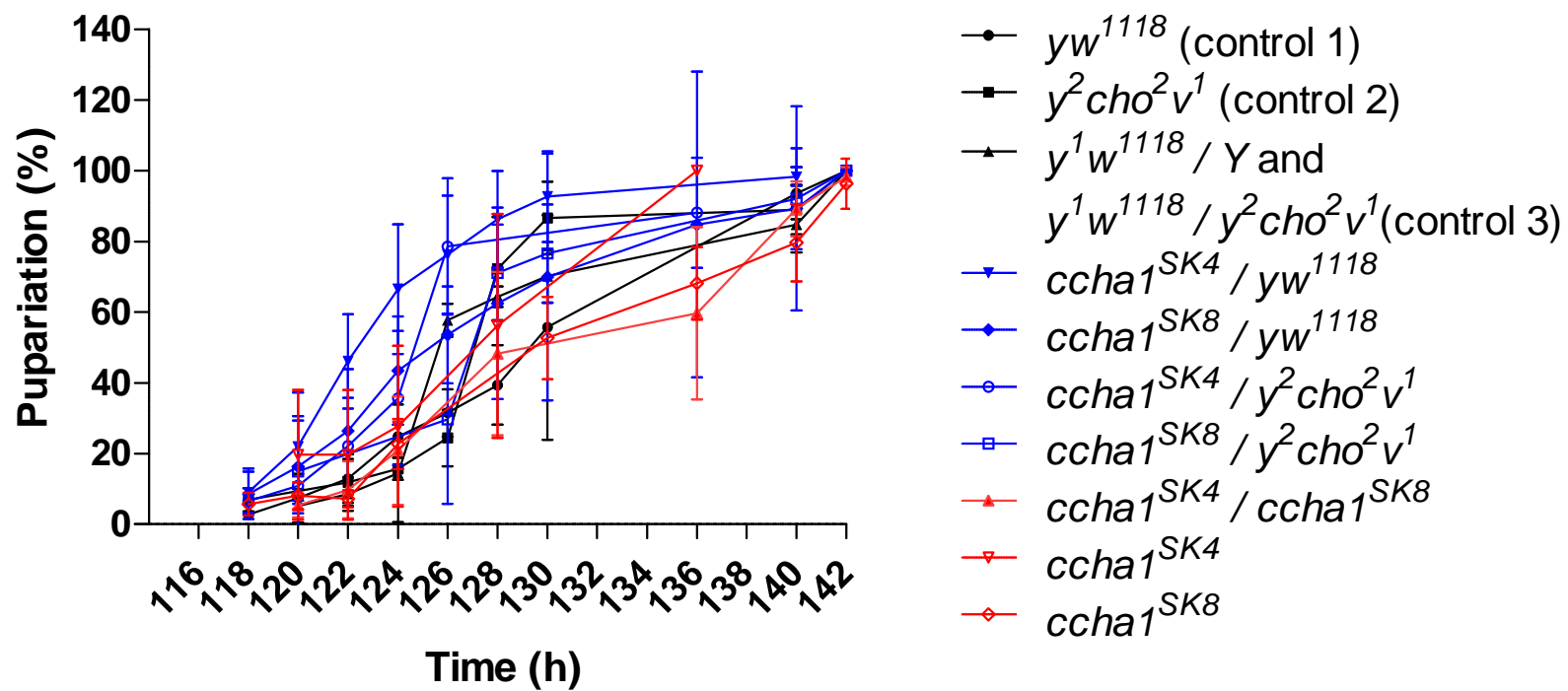

Fig. S2.

Supplement: S2 Fig — There is no difference between the time points, where the ccha1 mutants pupariate (127 hrs) and that of the control animals (ANOVA test, no significant difference). (PDF) [file pone.0133017.s002.pdf]
