# Supplementary material for: CCHamide-2 Is an Orexigenic Brain-Gut Peptide in Drosophila
Source: PLoS One. 2015 Jul 13;10(7):e0133017. doi: 10.1371/journal.pone.0133017 (PMC4500396; doi:10.1371/journal.pone.0133017)
Supplement: S1 Table — (PDF) [file pone.0133017.s003.pdf]

Table. S1. Primer list for qPCR

Gene	Sense	Antisense	Reference
RpLP0 (CG7490)	5’-ATCAAGGTTGTGGAACTGTTCG-3’	5’-GCGGGTTGTTCTCCAGATGA-3’	
RpL32 (CG7939)	5’-TAAGCTGTCGCACAAATGGCG-3’	5’-AACGCGGTTCTGCATGAGCA-3’	
RpL11 (CG7726)	5’-CGATCCCTCCATCGGTATCT-3’	5’-AACCACTTCATGGCATCCTC-3’	
dilp2 (CG8167)	5’-CAGGAGTTCGAGGAGGAGGA-3’	5’-AAGATAGCTCCCAGGAAAGAGG-3’	
dlip3 (CG14167)	5’-CAACGCAATGACCAAGAGAAC-3’	5’-GCATCTGAACCGAACTATCACTC-3’	
ccha2 (CG14375)	5’-AAACAGCAACAGCAGCAAAC-3’	5’-AGGACCACGGTGCAGATAAC-3’	[13]
ccha1 (CG14358)	5’-AGTGCAGTTGGACTTTGGTAGTGT-3’	5’-AGGGATGCTGTTTAGCATCTATGAC-3’	[21]
